# Accumulation of Heavy Metals in Roadside Soil in Urban Area and the Related Impacting Factors

**DOI:** 10.3390/ijerph15061064

**Published:** 2018-05-24

**Authors:** Meie Wang, Haizhen Zhang

**Affiliations:** 1 State Key Laboratory of Urban and Regional Ecology, Research Centre for Eco-environmental Sciences, Chinese Academy of Sciences, Beijing 100085, China; 2 Lingyin Administration Department (Hangzhou Flower Garden), Hangzhou Park and Cultural Relic Administration, Hangzhou 310012, China; haizhenzhang@hotmail.com

**Keywords:** urban soil, heavy metal contamination, traffic intensity, traffic emission

## Abstract

Heavy metal contamination in roadside soil due to traffic emission has been recognized for a long time. However, seldom has been reported regarding identification of critical factors influencing the accumulation of heavy metals in urban roadside soils due to the frequent disturbances such as the repair of damaged roads and green belt maintanance. Heavy metals in the roadside soils of 45 roads in Xihu district, Hangzhou city were investigated. Results suggested the accumulation of Cu, Pb, Cd, Cr, and Zn in roadside soil was affected by human activity. However, only two sites had Pb and Zn excessing the standards for residential areas, respectively, according to Chinese Environmental Quality Standards for soils. The concentrations of Cu, Pb, Cd, and Zn were significantly and positively correlated to soil pH and organic matter. An insignificant correlation between the age of the roads or vegetation cover types and the concentration of heavy metals was found although they were reported closely relating to the accumulation of heavy metals in roadside soils of highways. The highest Pb, Cd, and Cr taking place in sites with heavy traffic and significant differences in the concentrations of Cu, Pb, Cd, and Zn among the different categories of roads suggested the contribution of traffic intensity. However, it was difficult to establish a quantitative relationship between traffic intensity and the concentrations of heavy metals in the roadside soil. It could be concluded that impaction of traffic emission on the accumulation of heavy metals in roadside soils in urban area was slight and soil properties such as pH and organic matters were critical factors influencing the retention of heavy metals in soils.

## 1. Introduction 

Elevated heavy metal concentrations were observed in roadside soils in a regional investigation, which was found to be the third most important factor impacting the accumulation of heavy metals in soils after the proximity of a city or waste disposal area [1]. With the relocation of industrial areas from cities to sites where there is low density of population, vehicle emissions have become the primary source of heavy metals in the urban environment [2]. 

Lead was the earliest and most widely studied heavy metal contaminant in roadside soil because of its role as a gasoline additve. As early as the 1970s, equations were developed to estimate the accumulation of lead in roadside soil based on the distance from the road and the average daily traffic volume [3,4]. Later studies started to include other heavy metals, such as Cd, Zn, and Cu [5]. The wearing of tires and brake linings are the main vehicular source of Zn, Cd, and Cu. Karim et al. [6] found high levels of Cu and Zn in soils in both pre-monsoon and post-monsoon conditions in a region with high traffic congestion. Zhang et al. [7] identified that Cr, Cu, Zn, As, Cd and Pb in roadside soil along the Qinghai–Tibet highway were related to traffic emission and their concentration decreased exponentially with the distance from the road. Relationship between the distance to the road and the concentrations of heavy metals in roadside soils has been the most widely studied [8,9]. It was recognized that roadside contamination in soils caused by traffic did not extend much more than 20 m away from the road [10]. The distance influenced by runoff water from roads was within a range of 0–5 m, while areas beyond 10 m were usually affected by wind and airflow [11]. Sun et al. [12] found by the analysis of Pb isotopic compositions that parent material and vehicle exhaust were two major sources of Pb in topsoil at a distance of 0–7 m away from the road side, and 24.0% of the Pb come from vehicle exhaust. Besides, traffic burden is the other factor impacting the enrichment of heavy metals in roadside soil. Wang et al. [13] studied six highway segments on Tibetan Plateau and found that segment with higher traffic volume and greater proportion of high-emission vehicles showed greater levels of enrichment. They also found concentrations of Cr and Cu in roadside soil were significantly related with traffic volumes.

In spite of widely studying of the accumulation of heavy metals in roadside surface soil, most of those studies were conducted on highways. However, frequent disturbances of roadside soil by human activities in urban areas, such as the repair of damaged roads and green belt maintenance, make the accumulation of heavy metals in soil hard to predict. Besides, identification of factors influencing heavy metal accumulation in surface soil in urban area will be helpful in urban design and urban environmental management. Thus, in this work, we chose a scenic area in Hangzhou, China, as the studied area. The purpose of this work is to characterize the accumulation of heavy metals in roadside soil in urban areas and to evaluate impacts of those factors specific to urban environmental management for roads, such as traffic intensity, vegetation cover, and construction ages on the accumulation of heavy metals in soils.

## 2. Materials and Methods

### 2.1. Study Area

Xihu district (120°04′–120°10′E, 30°10′–30°16′N) is located in the south-west of Hangzhou city, China. It has an area of about 90 km^2^. More than 90% of the area belongs to the Xihu scenic area, in which natural landscapes such as mountains and lakes are the main topographic characteristic. No industrial activities are conducted around the scenic area and the porpotion of agricultural land use is small (no more than 5%). There is also a small urban area (less than 10% of the total area) in the east, northeast and north edge with land uses including residential and business areas. Thus, traffic emissions are almost the only pollution source of heavy metals in the area.

Three categories of roads according to their functions in this area were classified. They are scenic roads (SR) which are located within the scenic area, cross urban roads (CUR) which are used to connect between Hangzhou city and outside, and urban roads (UR) which are located within the urban area (in the east, northeast, and north of the study area). As for SRs, the traffic burden is the heaviest on weekends or holidays. High-emission vehicles such as heavy trucks mostly appear in CURs. The traffic volumes in URs are as normal as other urban area. 

Sampling sites were further classified into four groups according to the type of vegetation such as trees, trees and grass, trees and shrubs, and trees, shrubs, and grass in order to explain the impaction of vegetation on the accumulation of heavy metals in roadside soil.

### 2.2. Sampling

**Figure 1:**
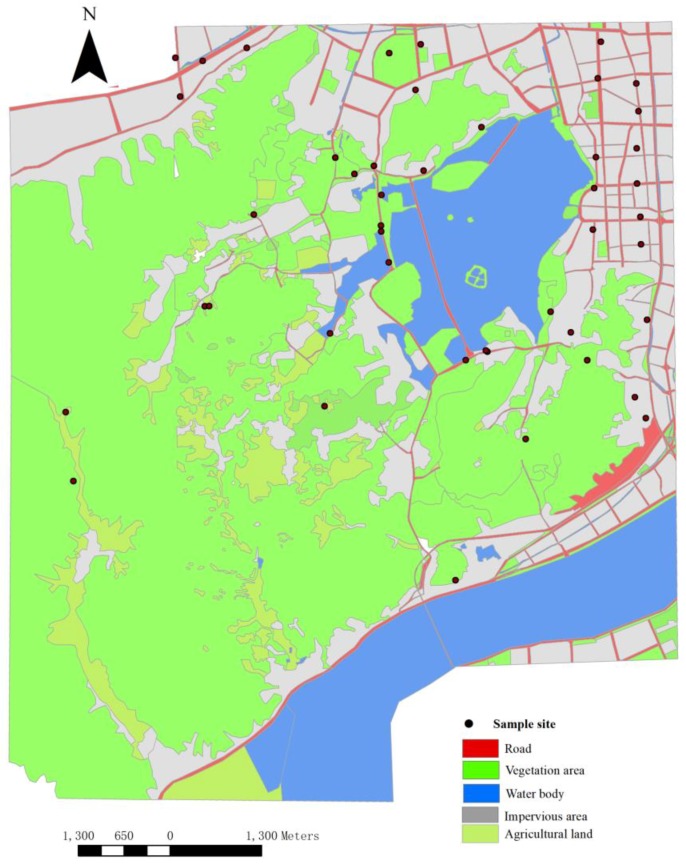
Map of sampling.

The spatial distribution of sample sites was shown in Figure 1. There were 18 sample sites for SR, 11 for CUR, and 16 for UR. Each sample was collected at the roadside within 2 m of the road edge at locations covered by vegetation. All sampling sites were located at intersections with traffic lights. A composite sample of surface soil (0–10 cm) was collected by mixing five subsamples within a 1 × 1 m^2^ square. Altogether, 45 samples were collected for 45 roads. Three roads for each group were chosen to estimate the traffic intensity by checking electronic recorders at the intersection. Daily traffic flows for a Monday to Saturday period in July and December, respectively, were chosen randomly. Two sets of data from 2010 and 2012 were used to obtain the average daily traffic intensity. 

### 2.3. Analysis of Soil Chemical and Physical Parameters

The total concentrations of nine heavy metals (Cu, Cr, Pb, Zn, Cd, Ni, Co, Mn and V) in soil samples were analyzed by four acid digestion method [14]. Briefly, a 0.25 g soil sample was digested with 10 mL HCl, 5 mL HNO_3_, 3 mL HF, and 3 mL HClO_4_ on the hotpot. The digested extract was then diluted to 50 mL with deionized water for subsequent measurement using Inductively Coupled Plasma Optical Emission Spectroscopy (ICP-OES, Prodigy7, Leemanlabs, Hudson, NH, USA) and Inductively Coupled Plasma Mass Spectroscopy (ICP-MS, NexION 300x, PerkinElmer, Waltham, MA, USA). A reference soil (GSS-5) from Institute of Geophysical and Geochemical Exploration, China was used as the quality control for the extract procedure. The detected concentrations for GSS-5 were (mg·Kg^−1^): Cu 141 ± 2.3, Cd 0.381 ± 0.02, Pb 568 ± 15.8, Zn 451 ± 11.6, Cr 109 ± 8.81, Ni 32.3 ± 0.73, Co 11.8 ± 2.2, V 152 ± 1.80, Mn 1368 ± 42.4. The known values of these metals in GSS-5 were (mg·Kg^-1^): Cu 144 ± 6, Cd 0.45 ± 0.06, Pb 552 ± 29, Zn 494 ± 25, Cr 118 ± 7, Ni 40 ± 4, Co 12 ± 2, V 166 ± 9, and Mn 1360 ± 7. Thus, the values detected were within the range of the standard. 

Soil organic matter was determined as reported by Wang et al. [14] that a soil aliquot was treated with 1 M HCl for 24 h and then detected for carbon content using the Elementar Vario ELIII (Elementar, Langenselbold, Germany). Soil pH was determined in a 1:2.5 (weight/volume) soil and water suspension. Soil texture was determined by a laser particle size analyzer, and the outcomes were reported according to the USDA (Department of Agriculture, Washington, DC, USA) soil classification scheme. 

### 2.4. Data Analysis

Descriptive statistics, an analysis of variance (ANOVA) and multiple comparison, normality analysis, and multivariate analysis (PCA) were conducted using SPSS 18.0 (SPSS Inc., IBM, Chicago, IL, USA). Descriptive statistics including frequency distribution, coefficient of variation (CV), normality test was conducted for the heavy metal concentrations of the 45 samples. An analysis of variance (ANOVA) and multiple comparison were conducted among groups of different traffic intensities and types of vegetation. Kaiser-Meyer-Olkin measure (KMO) of sampling adequacy and Bartlett’s test of sphericity were used to test the validity of PCA. There were 45 samples for nine elements, which basically satisfied the requirement of minimium sample size (amount of cases is more than five times of the amount of variables) [15]. Charts for the comparison of traffic intensity among the different categories of roads were produced using EXCEL 2010 (Microsoft, Redmond, WA, USA).

## 3. Results and Discussion

### 3.1. Descriptive Statistics and Comparison

The variation of heavy metal concentrations among the sampling sites was so large that five heavy metals (Cu, Zn, Pb, Cr and Cd) had a coefficient of variation (CV) of more than 90% (Table 1). The average concentrations of Cu, Zn, and Pb in roadside soil were higher than the corresponding geochemical background value in soil in Hangzhou [16]. Further Kolmogorov-Smirnov test for the normality of the data showed that only elements Mn, Co, and V demonstrated normal distribution, which indicated that the accumulation of these three metals was slightly disturbed by human activities. 

### 3.2. Multivariate Analysis

PCA based on correlation matrix was conducted. Both Kaiser-Meyer-Olkin measure of sampling adequacy (KMO = 0.589) and Bartlett’s test of sphericity (*p* = 0.000) indicated the validity of using PCA in this work. As shown in Table 2, altogether three factors could be grouped for the nine tested elements with total variance of 82.9%. Factor 1 includes Cu, Zn, Pb, and Cd and represents about 37.0% of the total variance. Factor 2 including Ni and Cr represents about 23.0% of the total variance. Factor 3 includes Co, V and Mn and represents about 22.0% of the total variance.

**Figure 2:**
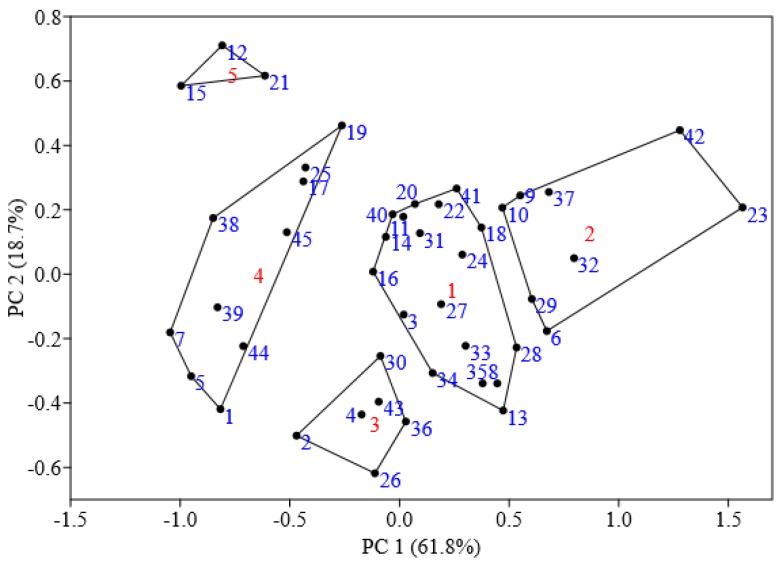
Grouping of sampling sites based on PCA.

Results of PCA also suggested that the 45 sampling sites could be divided into five groups (Figure 2). The first group was made up of 18 sampling sites (40% of the total amount of sites investigated). The cumulative percents of soil heavy metal concentrations on those sites ranged from 10 to 90% of each element’s dataset, which means soil heavy metal concentrations on sites of this group were at the median level. The second group was made up of 8 sites which has the highest concentrations of soil Pb and Cd, of which four sites are located in three busy scenic areas around the West Lake and the other four sites are located in four cross urban roads in the east-south part. The third group were made up of six sites with the lowest concentrations of V and Pb, while the fourth group including 10 sites had the lowest concentrations of Cd and Pb. The fifth group was made up of only three sites with the highest concentration of Cr, all located in busy business districts. 

### 3.3. Correlation between the Concentrations of Heavy Metals and Soil Properties

As shown in Table 3, the mean pH value in the studied area was 6.82, i.e., close to neutral. However, the pH values of the sampling sites ranged from 3.81 to 7.99, i.e., from strong acid to weak alkaline. The same large range was found for other soil properties such as the clay and organic concentrations. The highest levels of clay and organic carbon were almost 10 times the lowest values.

As shown in Table 4, the concentrations of Cu, Zn, and Pb were significantly and positively correlated to soil pH values (*p* < 0.01). A markedly significant and positive correlation between the organic matter concentration and the concentrations of Cu, Zn, Pb, and Cd was also found (*p* < 0.01). There was a significant positive correlation between clay and V concentration (*p* < 0.01). Soldi et al. [17] found the concentration of V was higher in a soil profile in the presence of clay than that in the presence of sand because clay would decrease the permeability and thus prevent the migration of the metal. Our results also showed that the retention of V in roadside soil in the study area was correlated with the clay content.

### 3.4. The Influence of Road Types on the Concentration of Heavy Metals in Roadside Soil 

Twelve roadside soil samples were collected from roads constructed less than 30 years ago, nine samples were collected from roads constructed 30 to 60 years ago, and 24 samples were collected from roads constructed more than 60 years ago. However, only the Cr, Co, and Ni concentrations in the samples collected from roads constructed 30 to 60 years ago were significantly higher than the other two age groups. 

Results of ANOVA and multiple comparison showed that the trees and shrubs group had a significantly higher concentration of Cr, Ni, Cu and Zn compared to the other groups. The tree, shrub, and grass group had a higher Pb concentration than the other groups. Less soil management is made for roadside green land with shrubs compared with that with trees only or trees and grass, which might lead to relatively high accumulation of heavy metals in roadside soils with shrubs. 

**Figure 3:**
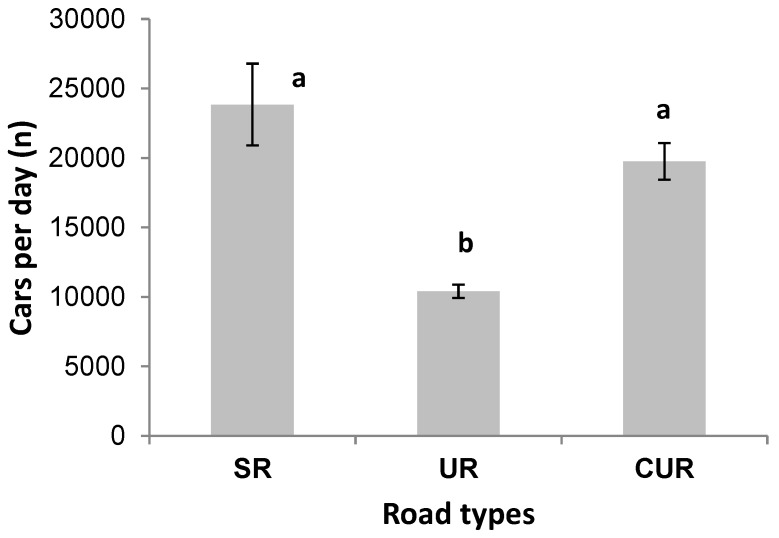
Comparison of traffic intensity among scenic roads (SR), urban roads (RD), and cross urban roads (CUR). Letters (a, b) refer to the difference at significance level of 0.05 (*p* < 0.05).

As shown in Figure 3, the average daily traffic intensity of urban road (UR) was about 10,400 cars per day, which was lower than that of scenic road (SR) (23,840 cars per day) and cross urban road (CUR) (19,748 cars per day). Accordingly, as shown in Table 5, CURs have the highest concentrations of Cu, Zn, Pb, and Cd among the three road types. However, the differences in concentrations of Cu, Pb, and Cd between CUR and SR were insignificant. For Zn, the differences between SR and UR were insignificant. UR had significantly higher concentrations of Cr and Co than those in SR.

## 4. Discussion

### 4.1. Identification of Heavy Metal Pollutants in Roadside Soils of the Studied Area

Our previous study of the heavy metal distribution in the urban soils in Beijing suggested that the longer the period of urbanization the greater the concentrations of Cu, Cd, Pb, and Zn in soils [18]. The investigation in Beijing city also found that the concentrations of Cd, Pb, Cu, and Zn decreased with increasing distance from the road [19]. Common anthropogenic sources of Cu, Pb, and Zn (i.e., vehicle emissions) were reported in an investigation of urban soils in Karachi [6]. As early as the 1990s, a study based on the concentration profiles of heavy metals in forest soils beside a busy urban road, showed that the concentration of Pb and Cd in the soil at the edge of the road was five times higher than in forest soil far from the road [20]. Elements Cr and Ni are mostly found in the same group because they are usually from the same source. There are several reports about the accumulation of Cr and Ni in roadside soil. For example, Chen et al. [19] did not find that concentrations of Cr and Ni in roadside soil were as elevated as those of Cu, Cd, Pb, and Zn. The same result was obtained by Werkenthin et al. [11], who synthesized roadside metal concentration data in Europe from the published literature. Munch [20] reported a two-fold increase of Cr and Ni concentrations in roadside soil compared to natural soil. Cobalt is often considered to be a marker of the parent material. Vanadium is a transitional trace element and accumulated readily in soils following the intense weathering of rocks and by the mobilization of elements. Vanadium is also widely used in refineries. In the environment, vanadium has been reported to be related to domestic heating and vehicle emissions [17]. As shown in Table 1, the average concentration of V was lower than the geochemical background. We assumed that the specific geochemical properties rather than the source of V enabled it to be separated from other elements. High soil Mn makes it difficult to impact its accumulation significantly by human activities except for strong sources such as mining and smelting emission. Normality analysis above showed little anthropogenic disturbance to the accumulation of Mn in this work, either. 

Accordingly, the accumulations of heavy metals Cu, Cd, Pb, Zn, and Cr in roadside soils in this work were impacted by human activities based on the multivariate statistics analysis and comparison with the background values. However, as suggested by Chinese Environmental Quality Standards for Soils, the standards of Cu, Cd, Pb, Zn, and Cr for soils in broadly speaking residential areas (including parks, residential blocks, schools, green lands etc.) are 300, 10, 300, 500, and 400 mg·Kg^−1^ [21]. Only two sites had Pb and Zn concentration excessed the standard 300 and 500 mg·Kg^-1^, respectively in this work (Table 1). Thus, it could be included that there was no heavy metal pollution in this studied area in general.

### 4.2. Factors Impacting the Accumulation of Heavy Metals in Roadside Soils

Soil properties including pH and organic matter content, prevailing wind direction, vegetation cover, and traffic intensity, etc. are important factors impacting the accumulation of heavy metals in soils [9,10,19]. The study by Kocher et al. [22] showed that the pH in roadside soil would increase to neutral or even above neutral levels due to road abrasion. An increased pH was reported to increase the adsorption of metals by competition between protons and metal cations and by the increase in the solubility of organic matter [11]. Soil organic matter was considered as the other important factor contributing the retention of heavy metals in roadside soils [5,23]. It was found by Turer et al. [5] that vehicle emissions and asphalt paving materials were main sources of insoluble organic matter in roadside soils and Pb, Zn, and Cu were largely concentrated in the insoluble organic fraction. In our investigation, the significance of pH and soil organic matter explained almost 100% of the variance of the accumulation of Cu, Zn, Pb, and Cd in roadside soil, which means that the pH and organic matter were two important factors influencing the retention of heavy metals in roadside soil.

The grouping of sampling sites based PCA suggested that the highest Pb, Cd, and Cr took place in the places where the traffic burdens were heavy. The higher concentrations of Pb, Cu, Cd, and Zn in the roadside soil of CUR compared with other two types of roads might also be related to the heavy traffic intensity and the burden of high-emission vehicles. Effects of traffic volumes would be complicated by other factors, such as the age of the road, direction and speed of wind, amount of rainfall, and soil properties, which all had direct influences on metal concentrations [24]. Overall, there was a trend for higher heavy metal roadside concentrations in locations where traffic intensity is higher [25,26]. However, unlike those studies conducted in highways, roadside soils in urban area are usually involved with much more complicated factors. For example, it was found from field observations in this work that most of the sites in the trees and shrubs, and trees, shrubs, and grass vegetation groups were alongside roads with an age of 30 to 60 years, and also fell into the UR category. Thus, those factors considered to impacting the accumulation of heavy metals significantly in roadside soils in highways, such as construction age, vegetation, and even vehicle volume were less apparent for urban environment. 

## 5. Conclusions

Heavy metal accumulation in roadside soils from traffic emission is one of the significant urban environmental issues, which is critical for environmental management. In this study, we collected roadside soil samples from 45 roads in three different categories in Xihu district, Hangzhou city, and investigated the potential factors impacting the concentrations of heavy metals. Our results showed that

● CVs larger than 90% were found for Cu, Zn, Pb, Cr, and Cd. The concentrations of Cu, Zn, and Pb were elevated compared to the corresponding geochemical background values. Clear divisions among the heavy metals were also identified following a multivariate analysis. It could be concluded that Cu, Zn, Pb, Cr, and Cd in roadside soils in the studied area might have accumulated due to human activity, while no heavy contamination was suggested based on Chinese Environmental Quality Standards for soils.
● It was suggested by correlation analysis that pH and soil organic matter were the two most significant factors influencing the retention of heavy metals in roadside soil, because those two factors could explain almost 100% of the variance of the accumulation of Cu, Zn, Pb, and Cd.
● Slight correlation was found between the age of the roads or the type of roadside vegetation cover and concentrations of the main anthropogenic heavy metals (Cu, Pb, Cd, Cr, and Zn), though those factors have been widely considered to impact the accumulation of heavy metals in roadside soils in highways. However, the highest Pb, Cd, and Cr taking place in sites with heavy traffic and significant differences in the concentrations of Cu, Pb, Cd, and Zn among the different categories of roads suggested the contribution of traffic intensity. The CUR with the highest traffic volumes had the highest concentrations of Cu, Zn, Pb, and Cd compared with the other two road categories.

## Figures and Tables

**Table 1:** Descriptive statistics for each metal and a comparison with the geochemical background value (mg·Kg^−1^).

Elements	n	Min	Max	Mean	SD	CV (%)	K-S Test
Mn	45	92.5	770	449	114	25.4	0.200
Backgroud	1452			609	109	18	
Cu	45	2.17	198	38.7	36.1	93.3	0.000
Backgroud	1353			30.9	4.98	16	
Zn	45	43.2	885	139	131	94.2	0.000
Backgroud	1277			92.7	8.91	10	
Pb	45	2.13	346	70.0	68.2	97.4	0.000
Backgroud	1420			30.4	3.89	13	
Cr	45	20.6	255	53.3	49.5	92.9	0.000
Backgroud	1326			77.6	7.28	9	
Ni	45	6.65	75.1	22.9	13.4	58.5	0.000
Backgroud	1535			32.5	4.37	13	
Co	45	1.30	15.7	7.32	3.53	48.2	0.068
Backgroud	1396			13.8	1.56	7	
V	45	27.3	116	48.6	17.5	36.0	0.200
Backgroud	1358			99.4	7.36	7	
Cd	45	0.03	2.41	0.387	0.419	108	0.000
Backgroud	1382			1.52	0.267	18	

**Table 2:** Grouping of tested elements based on multivariate analysis (PCA).

Elements	Factor 1	Factor 2	Factor 3
Cr	−0.105	0.943	0.166
Co	−0.072	0.271	0.899
Ni	-0.013	0.911	0.160
Cu	0.972	−0.018	0.082
Zn	0.940	0.024	−0.084
V	−0.045	0.264	0.870
Pb	0.760	−0.209	0.132
Mn	0.217	−0.399	0.580
Cd	0.928	−0.051	−0.093
Variance %	37.0	23.0	22.0
Total eigenvalues	3.33	2.07	1.98

**Table 3:** The pH values and clay and organic carbon concentration in roadside soil in Xihu district, Hangzhou, China.

Items	pH	Clay (%)	Organic Matter (mg·Kg^−1^)
n	27	25	27
Mean	6.82	22.2	65.2
Range	3.81–7.99	0.65–59.4	13.1–128

**Table 4:** Spearman correlations between the concentrations of heavy metals and soil properties.

Parameters	Mn	Cu	Zn	Pb	Cr	Ni	Co	V	Cd
pH	−0.082	0.652 ^**^	0.596 ^**^	0.585 ^**^	0.225	−0.046	−0.139	−0.274	0.234
Clay	0.253	−0.175	−0.223	−0.023	0.283	0.254	0.268	0.626 ^**^	0.223
OM	−0.007	0.524 ^**^	0.541 ^**^	0.517 ^**^	0.025	−0.004	−0.321	−0.001	0.715 ^**^

^**^ refers to the significant level at *p* < 0.01.

**Table 5:** Comparison of heavy metal concentrations among scenic roads (SR), cross urban roads (CUR), and urban roads (UR).

Roads	n	Cr	Co	Ni	Cu	Zn	V	Pb	Mn	Cd
SR	18	39.0 ± 14.5	11.1 ± 2.97	24.0 ± 6.30	39.9 ± 19.6	133 ± 51.1	50.6 ± 22.1	76.3 ± 71.4	470 ± 133	0.477 ± 0.209
		b	b	a	ab	b	a	ab	a	a
CUR	11	59.9 ± 27.9	13.7 ± 4.31	27.1 ± 7.47	65.0 ± 58.5	234 ± 237	61.1 ± 21.7	112 ± 85.9	457 ± 87.0	0.580 ± 0.735
		ab	ab	a	a	a	a	a	a	a
UR	16	77.4 ± 75.3	13.7 ± 3.70	33.2 ± 21.6	31.5 ± 19.1	92.3 ± 37.7	56.4 ± 16.4	51.8 ± 45.	463 ± 113	0.158 ± 0.135
		a	a	a	b	b	a	b	a	b

Letters (a, b) refer to the difference at significance level of 0.05 (*p* < 0.05).
